# Genetic effect of *MTHFR* C677T, A1298C, and A1793G polymorphisms on the age at onset, plasma homocysteine, and white matter lesions in Alzheimer's disease in the Chinese population

**DOI:** 10.18632/aging.202827

**Published:** 2021-04-04

**Authors:** Yaling Jiang, Xuewen Xiao, Yafei Wen, Meidan Wan, Lu Zhou, Xixi Liu, Xin Wang, Lina Guo, Hui Liu, Yafang Zhou, Junling Wang, Xinxin Liao, Lu Shen, Bin Jiao

**Affiliations:** 1 Department of Neurology, Xiangya Hospital, Central South University, Changsha, China; 2 Department of Geriatrics Neurology, Xiangya Hospital, Central South University, Changsha, China; 3 National Clinical Research Center for Geriatric Disorders, Central South University, Changsha, China; 4 Key Laboratory of Hunan Province in Neurodegenerative Disorders, Central South University, Changsha, China; 5 Key Laboratory of Organ Injury, Aging and Regenerative Medicine of Hunan Province, Changsha, China

**Keywords:** *MTHFR*, Alzheimer's disease, homocysteine, white matter lesions, Chinese

## Abstract

Background: Three polymorphisms in the *Methylenetetrahydrofolate reductase (MTHFR)* gene (C677T, A1298C, and A1793G) were reported associated with AD. However, their genotype distributions and associations with age at onset (AAO), homocysteine, and white matter lesions (WML) were unclear in the Chinese AD population.

Method: We determined the presence of C677T, A1298C, and A1793G polymorphisms in the *MTHFR* gene using Sanger sequencing in a Chinese cohort comprising 721 AD patients (318 early-onset AD patients (EOAD) and 403 late-onset AD patients (LOAD)) and 365 elderly controls. Additionally, the homocysteine level and WML were evaluated in 121 AD patients.

Results: The frequency of allele T of C677T polymorphism was significantly higher in AD patients than in controls (*P* = 0.040), while no statistical difference was observed in A1298C and A1793G (*P* > 0.05). Besides, genotype distributions of C677T and A1298C polymorphisms statistically varied between AD patients and controls (*P* = 0.021, *P* = 0.012). Moreover, the AAO was significantly lower in CT/TT (C677T) genotypes carriers (*P* = 0.042) and higher in AC/CC (A1298C) and AG/GG (A1793G) genotypes carriers (*P* = 0.034, *P* = 0.009) in patients with LOAD. We also found that patients with CT/TT (C677T) genotypes were prone to present an increased homocysteine level (*P* = 0.036) and higher Fazekas score (*P* = 0.024). In comparison, patients with AG/GG genotypes (A1793G) had a significantly lower Fazekas score (*P* = 0.013).

Conclusions: The genotype distributions of C677T and A1298C polymorphisms are associated with AD in the Chinese population. Moreover, AD patients with C677T polymorphism are prone to present an earlier onset, higher homocysteine level, and more severe WML.

## INTRODUCTION

Alzheimer's disease (AD) is a progressive neurodegenerative disease. It is the most common form of dementia in the elderly, mainly characterized by the progressive decline in memory and cognitive function. AD can be classified as early-onset AD (EOAD, age at onset (AAO)<65 years) and late-onset AD (LOAD, AAO≥65 years). The etiology of AD is multifactorial and complex. Mutations in the *amyloid precursor protein* gene *(APP)*, *presenilin1* gene *(PSEN1)*, and *presenilin2* gene *(PSEN2)* are the leading causes for familial EOAD. Simultaneously, the convergence of genetic and environmental factors in aging is the primary drive for sporadic LOAD. Among multiple genetic factors associated with sporadic LOAD, *apolipoprotein E (APOE)* is the most critical risk factor [1, 2]. To date, genome-wide association studies have identified more than 50 AD-related genes/loci, shedding new light on the pathogenesis of AD [3].

It is well established that the *Methylenetetrahydrofolate reductase* (*MTHFR*) gene is critical for the folate cycle and homocysteine metabolism. Reduction and loss of MTHFR function lead to an elevated homocysteine level, which is considered a risk factor for AD. In addition, Hyperhomocysteinemia has also been linked to white matter signal abnormalities (WMSA) in magnetic resonance imaging (MRI), which is also recognized as a risk factor for AD [4, 5]. Recently, Hoffman *et al*. [6] proposed a novel mechanism leading to AD. The genetic MTHFR deficiency could enhance phosphorylation of amyloid-β protein precursor (AβPP) at Thr668, correlating with enhanced accumulation of demethylated protein phosphatase 2A (PP2A) and activation of glycogen synthase kinase-3β (GSK-3β), which was known to critically influence neuronal AβPP function and pathological amyloidogenic processing [6].

So far, three polymorphisms (C677T (rs1801133), A1298C (rs1801131), and A1793G (rs2274976)) of the *MTHFR* gene have been reported to be associated with AD. Among them, the C677T is the most significant polymorphism with most investigations in the clinic. The T allele prevalence (C677T) varied across ethnic groups and regions, with a range from 12% to 57% in European, Asian, American, and African populations [7, 8]. It should be pointed out that the C677T polymorphism was associated with increased susceptibility of AD in Asian and Caucasian populations [9–12]. However, there is still controversy over whether A1298C is related to the risk of AD. Previous studies have proposed that the A1298C polymorphism might play a protective role in developing AD in Indian and Japanese populations [13, 14], which was inconsistent with the Tunisian population's results reported by Mansouri *et al*. [15]. As for A1793G polymorphism, the protective effect was only confirmed in Japanese AD patients [13]. In the present study, we aimed to explore the genotype distributions of three polymorphisms and their associations with age at onset (AAO), homocysteine, and white matter lesions (WML) in the Chinese AD population.

## MATERIALS AND METHODS

### Study subjects

A total of 721 patients with AD and 365 cognitively unimpaired control subjects were involved in the study (Table 1). AD patients were recruited from the Department of Neurology, Xiangya Hospital, Central South University (Hunan, China). All patients were clinically diagnosed by two experienced neurologists and met the National Institute of Neurological and Communicative Disorders and Stroke and Alzheimer's Disease and Related Disorders Association criteria [16] for probable or definite AD. Among them, 121 AD patients performed homocysteine examinations and brain MRI scanning. Besides, 365 unaffected community-dwelling individuals without AD symptoms were recruited as healthy controls and matched for ethnicity and area of residence. The study was approved by the Ethics Committee of Xiangya Hospital, Central South University (institutional review board equivalent). Written informed consent was obtained from all participants involved in the study.

**Table 1:** Basic information of subjects.

	**AD**	**Controls**	***P*-value**
**Numbers**	721	365	
**Age (years)**	65.80±10.91	70.65 ± 5.35	0.000
**Gender**			
Female	426 (59.1%)	190 (52.1%)	0.027
Male	295 (40.9%)	175 (48.0%)	
**MMSE**	10.96 ± 7.30	27.79 ± 1.51	0.000
***APOE4***			
*APOE4* non-carriers	409 (56.7%)	293 (80.3%)	0.000
*APOE4* carriers	312 (43.3%)	72 (19.7%)	

### Genetic testing

Blood samples (10 mL per subject) were obtained by venipuncture from each subject and transferred to ethylenediaminetetraacetic acid (EDTA) tubes. Genomic DNA was isolated from peripheral blood leukocytes using a standard protocol [17]. All isolated DNA samples were measured for quality and quantity by a fluorometer and normalized to 50 ng/μL. Polymerase chain reaction (PCR) was performed on *MTHFR* (NM_001330358) C677T, A1298C, and A1793G polymorphisms. Primers for *MTHFR* and *APOE* genotypes amplification were shown in Supplementary Table 1. All PCR products were sequenced with Big Dye terminator v3.1 sequencing chemistry on an ABI 3730xl DNA analyzer (Applied Biosystems). DNA sequences were analyzed using the sequencing software of Mutation Surveyor (Softgenetics). Variants were checked against established databases (Genome Aggregation Database (gnomAD) and dbSNP v.150).

### Homocysteine examinations

Venous blood samples were collected using standard venipuncture protocols after an overnight fast. The total plasma homocysteine level (umol/L, reference 0–20 umol/L) was measured by high-performance liquid chromatography within 3 hours of blood collection.

### MRI data acquisitions and Fazekas score

Magnetic resonance imaging (MRI) data were acquired in a 3.0T Philips Achieva system (Philips Medical Systems), using a 3D T1-weighted Turbo Field Echo (TFE) sequence (RT = 7.2 ms, ET = 3.2 ms, flip angle=9°, number of slices=160, matrix size=250 × 250 mm, slice thickness=1.0 mm); and a 3D FLAIR sequence (RT = 48000 ms, ET = 280 ms, TI = 1650 ms, flip angle=90°, number of slices=140, matrix size=250 × 237 mm, slice thickness=2.0mm).

The Fazekas scale is a widely used method to visually rate hyperintense white matter signal abnormalities (WMSA) in MRI data. The Fazekas scale was applied on FLAIR MRI data on the axial plane and was scored following standard guidelines [18]. Briefly, Fazekas grades WMSA as 0 (i.e. absence of WMSA), 1 (i.e. punctate WMSA), 2 (i.e. early confluent WMSA), and 3 (i.e. WMSA in large confluent areas).

### Statistical analyses

Descriptive statistics were described as the mean ± standard deviation. The Mann–Whitney U test was used to compare age and Mini-Mental State Examination (MMSE). The chi-square test was used to compare gender and the distribution of alleles and genotypes of *MTHFR* polymorphisms between AD patients and controls. The Bonferroni method was used to perform the pairwise test between multiple groups based on the chi-square test. Each subscript letter denotes a subset of intervention categories whose row/column proportions do not differ significantly from each other at the 0.05 level. For example, the group marked 'a' is not statistically significant from the other group marked 'a' (*P* > 0.05), and the group marked 'a' is statistical significantly from the group marked 'b' (*P* < 0.05). The Kruskal-Wallis test was used to confirm the significant difference in the AAO and homocysteine levels among different genotypes in AD patients. The chi-square test was used to determine the significant difference of Fazekas score among different genotypes in AD patients. The statistical analyses were performed by SPSS version 23.0. *P* < 0.05 was considered statistically significant.

## RESULTS

A total of 721 patients with AD and 365 cognitively unimpaired control subjects were involved in the study. The mean age of the AD group was 65.80 ± 10.91 years, and the proportion of females was 59.1%. The control group's mean age was 70.65 ± 5.35 years, and the ratio of females was 52.1%. In addition, the mean score of MMSE in AD patients was 10.96 ± 7.30, while in controls was 27.79 ± 1.51 (*P* = 0.000). The frequency of *APOE4* carriers in AD patients (43.3%) was significantly higher than in controls (19.7%, *P* = 0.000) (Table 1). Moreover, 318 cases (44.1%) of the 721 patients were diagnosed as EOAD (AAO < 65) and 403 cases (55.9%) were LOAD (AAO ≥ 65). Among them, 121 AD patients performed homocysteine examinations and MRI data acquisition.

### Distributions of *MTHFR* C677T, A1298C, and A1793G polymorphisms in AD patients and controls

The allele and genotype distributions of *MTHFR* C677T, A1298C, and A1793G polymorphisms of all participants were presented in Table 2. Allele T of the C677T polymorphism was significantly more frequent in the AD group (29.7%) than the control group (25.5%, *P* = 0.040). Moreover, the genotype distribution showed a significantly higher TT genotype in AD patients (6.1%) compared with controls (2.5%, *P* < 0.05). In terms of the A1298C polymorphism, allele C was more common in the AD group (19.0%) than the control group (16.7%), but no statistical difference was determined (*P* > 0.05). Furthermore, the distribution of genotypes showed a significantly higher percentage of CC genotype in the AD group (2.4%) compared with the control group (0.0%, *P* < 0.05). However, as for the A1793G polymorphism, no statistical difference was observed in the allele and genotype distributions between AD patients and controls (*P* > 0.05) (Table 2).

**Table 2:** Allele and genotype distributions of *MTHFR* polymorphisms in AD patients and controls.

	**Total**	**C677T allele (%)**		**C677T genotype (%)**			**A1298C allele (%)**		**A1298C genotype (%)**			**A1793G allele (%)**		**A1793G genotype (%)**		
		**C**	**T**	**CC**	**CT**	**TT**	**A**	**C**	**AA**	**AC**	**CC**	**A**	**G**	**AA**	**AG**	**GG**
AD	721	1014 (70.3)	428 (29.7)	337 (46.7)^a^	340 (47.2)^a^	44 (6.1)^b^	1168 (81.0)	274 (19.0)	464 (64.4)^a^	240 (33.3)^a^	17 (2.4)^b^	1292 (89.6)	150 (10.4)	574 (79.6)^a^	144 (20.0)^a^	3 (0.4)^a^
Controls	365	544 (74.5)	186 (25.5)	188 (51.5)^a^	168 (46.0)^a^	9 (2.5)^a^	608 (83.3)	122 (16.7)	243 (66.6)^a^	122 (33.4)^a^	0 (0.0)^a^	665 (91.1)	65 (8.9)	300 (82.2)^a^	65 (17.8)^a^	0 (0.0)^a^
*P*-value		0.040		0.021			0.192		0.012			0.269		0.196		

a, b: each subscript letter denotes a subset of intervention categories whose row proportions do not differ significantly from each other at the 0.05 level (Bonferroni method).

The genotype distribution of the *MTHFR* gene in AD patients and controls was also determined after stratification of all samples by *APOE4* status. In *APOE4* carriers, the frequency for the T allele (C677T) was prominent in cases compared with controls (*P* = 0.039), and the distribution of the C677T genotypes was also significantly different (*P* = 0.047). At the same time, no statistical difference was determined in the A1298C and A1793G polymorphisms (*P* > 0.05). In *APOE4* non-carriers, allele and genotype distributions of the A1298C polymorphism were significantly different between AD patients and controls (*P* = 0.044, *P* = 0.003, separately). Still, no statistical difference was found in C677T and A1793G polymorphisms (*P* > 0.05) (Table 3).

**Table 3:** Allele and genotype distributions of *MTHFR* polymorphisms in *APOE4* carriers and non-carriers.

	**Total**	**C677T allele (%)**		**C677T genotype (%)**			**A1298C allele (%)**		**A1298C genotype (%)**			**A1793G allele (%)**		**A1793G genotype (%)**		
		**C**	**T**	**CC**	**CT**	**TT**	**A**	**C**	**AA**	**AC**	**CC**	**A**	**G**	**AA**	**AG**	**GG**
***APOE4* carriers**																
AD	312	431 (69.1)	193 (30.9)	139 (44.6)^a^	153 (49.7)^a^	20 (9.4)^a^	522 (83.7)	102 (16.2)	212 (67.9)^a^	98 (31.4)^a^	2 (0.6)^a^	569 (91.2)	55 (8.8)	258 (82.7)^a^	53 (17.0)^a^	1 (0.3)^a^
Controls	72	112 (77.8)	32 (22.2)	41 (56.9)^a^	30 (41.7)^a^	1 (1.4)^a^	120 (83.3)	24 (16.7)	48 (66.7)^a^	24 (33.3)^a^	0 (0.0)^a^	131 (91.0)	13 (9.0)	59 (81.9)^a^	13 (18.1)^a^	0 (0.0)^a^
*P*-value		0.039		0.047			0.925		0.634			0.935		0.795		
***APOE4* non-carriers**																
AD	409	583 (71.3)	235 (28.7)	198 (48.4)^a^	187 (45.7)^a^	24 (5.9)^b^	646 (79.0)	172 (21.0)	252 (61.6)^a^	142 (34.7)^a^	15 (3.7)^b^	723 (88.4)	95 (11.6)	316 (77.3)^a^	91 (22.2)^a^	2 (0.5)^a^
Controls	293	432 (73.7)	154 (26.3)	147 (50.2)^a^	138 (47.1)^a^	8 (2.7)^a^	488 (83.3)	98 (16.7)	195 (66.6)^a^	98 (33.4)^a^	0 (0.0)^a^	534 (91.1)	52 (8.9)	241 (82.3)^a^	52 (17.7)^a^	0 (0.0)^a^
*P*-value		0.312		0.145			0.044		0.003			0.098		0.110		

a, b: each subscript letter denotes a subset of intervention categories whose column proportions do not differ significantly from each other at the 0.05 level (Bonferroni method).

### Correlations of age at onset (AAO) with *APOE4* or *MTHFR* polymorphisms in AD patients

**Figure 1:**
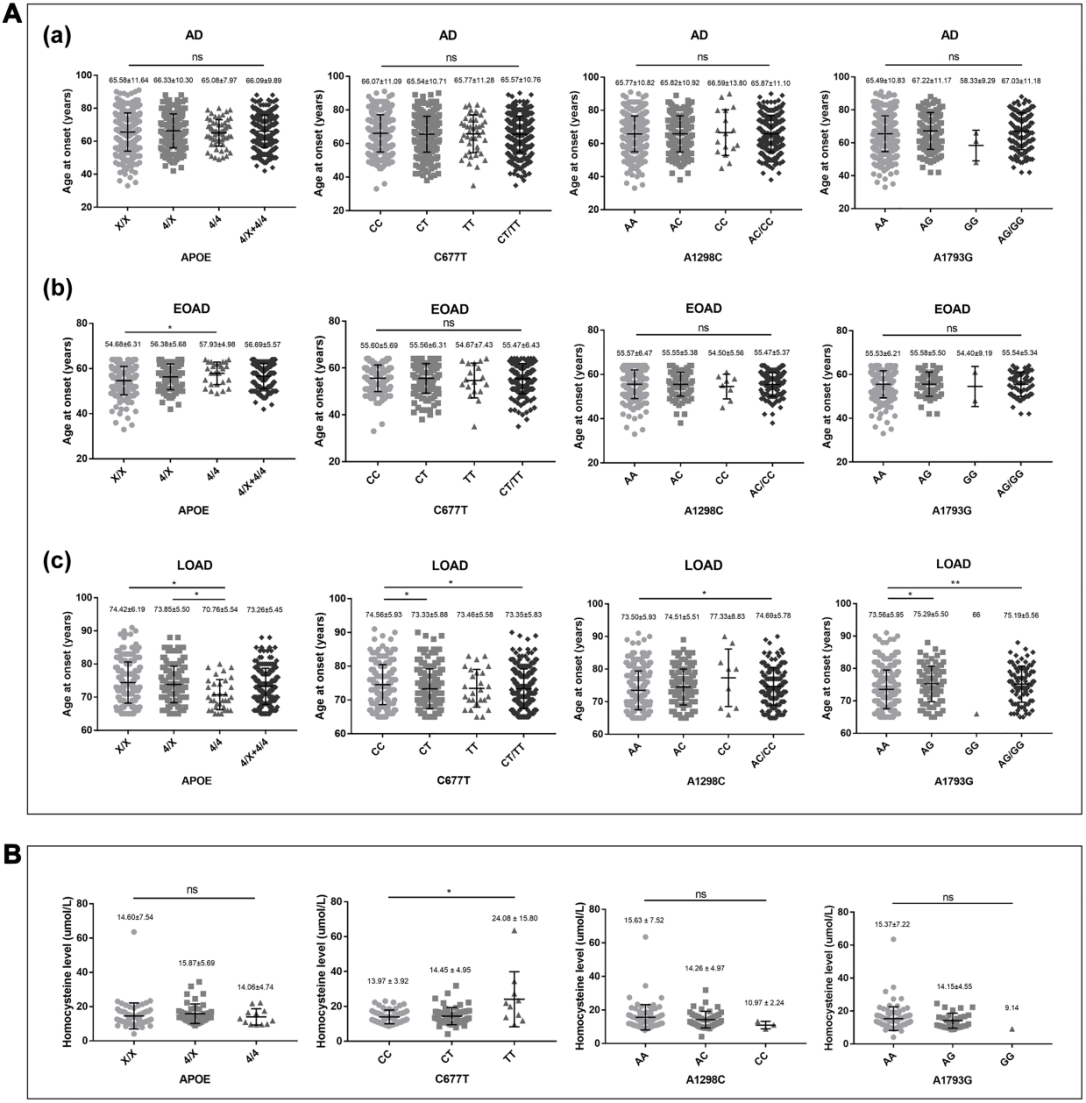
**Correlations of AAO and homocysteine with *APOE4, MTHFR* C667T, A1298C, and A1793G genotypes in AD patients.** (**A**) Correlation of AAO with *APOE4, MTHFR* C667T, A1298C, and A1793G genotypes in (a) AD (*N* = 721), (b) EOAD (*N* = 318), and (c) LOAD (*N* = 403). (**B**) Correlation of homocysteine with *APOE4, MTHFR* C667T, A1298C, and A1793G genotypes in AD (*N* = 121). Descriptive statistics were described as the mean ± standard deviation. Statistics performed by Kruskal-Wallis test, ^*^p < 0.05, ^**^p < 0.01; ns: no statistical difference.

After stratification of AD patients by AAO (EOAD (< 65 years) and LOAD (≥ 65 years)), AAO was significantly lower in LOAD patients carrying *APOE* 4/4 genotypes compared to *APOE* X/X genotype (lowered by 3.7 years, *P* = 0.015). As for *MTHFR* C677T polymorphism, AAO was significantly lower in LOAD patients carrying *MTHFR* CT/TT (C677T) genotypes compared to CC genotype (lowered by 1.2 years, *P* = 0.042). In terms of the A1298C polymorphism, AAO was significantly higher in LOAD patients carrying AC/CC (A1298C) genotypes (74.69 ± 5.78 years) compared to AA genotype (73.50 ± 5.93 years, *P* = 0.034). A similar pattern was observed in the A1793G polymorphism. AAO was significantly higher in LOAD patients carrying AG/GG (A1793G) genotypes (75.19 ± 5.56 years) compared to AA genotype (73.56 ± 5.95 years, *P* = 0.009). Nevertheless, no statistical difference was determined in all AD patients or EOAD patients (*P* > 0.05). (Figure 1A)

### Correlations of homocysteine with *MTHFR* polymorphisms in AD patients

The average level of homocysteine was 15.02 ± 6.65 umol/L in the 121 AD patients. AD patients with *MTHFR* TT (C677T) genotype (24.08 ± 15.60 umol/L) presented a significantly higher level of homocysteine compared to the CC genotype (13.97±3.92 umol/L, *P* = 0.036). Additionally, AD patients with CT (C677T) genotype (14.45 ± 4.95 umol/L) had a higher level of homocysteine compared to the CC genotype, but no statistical difference was observed (*P* > 0.05). In AC/CC (A1298C) carriers, the average levels of homocysteine (14.26 ± 4.97 umol/L and 10.97 ± 2.24 umol/L, respectively) were lower in comparison with AA (15.63 ± 7.52 umol/L), but no statistical difference was determined (*P* > 0.05). Similar to A1298C, the average levels of homocysteine were lower in AG/GG (A1793G) carriers (14.15 ± 4.55 umol/L and 9.14 umol/L, respectively) compared to AA (15.37 ± 7.22 umol/L), but no statistical difference was confirmed (*P* > 0.05). (Figure 1B)

### Correlations of white matter lesions (WML) with *MTHFR* polymorphisms in AD patients

The Fazekas scale is a widely used method to visually assess white matter lesions (WML) in MRI data. The Fazekas score was significantly higher in AD patients carrying the *MTHFR* T allele (C677T) (*P* = 0.006), as well as TT (C677T) genotype (*P* = 0.024). In terms of the A1793G polymorphism, the Fazekas score was significantly lower in patients with AG/GG genotypes (*P* = 0.013). However, no statistical difference was determined in AD patients with A1298C polymorphism (*P* > 0.05). (Table 4)

**Table 4:** Correlations of WML with *MTHFR* C667T, A1298C and A1793G genotypes in AD patients (*N* = 121).

		**C677T allele (%)**		**C677T genotype (%)**			**A1298C allele (%)**		**A1298C genotype (%)**			**A1793G allele (%)**		**A1793G genotype (%)**		
		**C**	**T**	**CC**	**CT**	**TT**	**A**	**C**	**AA**	**AC**	**CC**	**A**	**G**	**AA**	**AG**	**GG**
Fazekas	0	9 (5.4)	1 (1.4)	4 (7.0)^a^	1 (1.9)^a^	0 (0.0)^a^	8 (4.2)	2 (4.0)	4 (5.4)^a, b^	0 (0.0)^b^	1 (33.3) ^a^	8 (3.8)	2 (6.3)	4 (4.4)^a^	0 (0.0)^a^	1 (100.0)^b^
	1	97 (57.7)	29 (39.2)	37 (64.9)^a^	23 (42.6)^a^	3 (30.0)^a^	96 (50.0)	30 (60.0)	35 (47.3)^a^	26 (59.1)^a^	2 (66.7)^a^	104 (49.5)	22 (68.8)	41 (45.6)^a^	22 (73.3)^b^	0 (0.0)^a, b^
	2	39 (23.2)	31 (41.9)	10 (17.5)^a^	19 (35.2)^a, b^	6 (60.0)^b^	58 (30.2)	12 (24.0)	23 (31.1)^a^	12 (27.3)^a^	0 (0.0)^a^	66 (31.4)	4 (12.5)	31 (34.4)^a^	4 (13.3)^a^	0 (0.0)^a^
	3	23 (13.7)	13 (17.6)	6 (10.5)^a^	11 (20.4)^a^	1 (10.0)^a^	30 (15.6)	6 (12.0)	12 (16.2)^a^	6 (13.6)^a^	0 (0.0)^a^	32 (15.2)	4 (12.5)	14 (15.6)^a^	4 (13.3)^a^	0 (0.0)^a^
*P* value		0.006		0.024			0.646		0.121			0.087		0.013		

a, b: each subscript letter denotes a subset of intervention categories whose column proportions do not differ significantly from each other at the 0.05 level (Bonferroni method).

### Gene-gene interactions of *MTHFR* and *APOE* in AD patients

The allele C of the A1298C polymorphism was significantly more frequent in the *APOE4* non-carriers (21.0%) than the *APOE4* carriers (16.3%, *P* = 0.025) in AD patients. And the genotype distribution showed a significantly higher CC genotype (3.7%) in *APOE4* non-carriers compared with *APOE4* carriers (0.7%, *P* < 0.05). In terms of the C677T and A1793G polymorphisms, there was a lack of association between *APOE4* and *MTHFR* polymorphisms in the Chinese AD population (*P* > 0.05). (Table 5)

**Table 5:** Gene-gene interactions of *MTHFR* and *APOE* in AD patients (*N* = 721).

	**C677T allele (%)**		**C677T genotype (%)**			**A1298C allele (%)**		**A1298C genotype (%)**			**A1793G allele (%)**		**A1793G genotype (%)**		
	**C**	**T**	**CC**	**CT**	**TT**	**A**	**C**	**AA**	**AC**	**CC**	**A**	**G**	**AA**	**AG**	**GG**
APOE4 carriers	431 (69.1)	193 (30.9)	139 (44.6)^a^	153 (49.0)^a^	20 (6.4)^a^	522 (83.7)	102 (16.3)	212 (67.9)^a^	98 (31.4)^a, b^	2 (0.7)^b^	569 (91.2)	55 (8.8)	258 (82.7)^a^	53 (17.0)^a^	1 (0.3)^a^
APOE4 non-carriers	583 (71.3)	235 (28.7)	198 (48.4)^a^	187 (45.7)^a^	24 (5.9)^a^	646 (79.0)	172 (21.0)	252 (61.6)^a^	142 (34.7)^a, b^	15 (3.7)^b^	723 (88.4)	95 (11.6)	316 (77.3)^a^	91 (22.2)^a^	2 (0.5)^a^
*P* value	0.822		0.588			0.025		0.014			0.084		0.195		

a, b: each subscript letter denotes a subset of intervention categories whose column proportions do not differ significantly from each other at the 0.05 level (Bonferroni method).

## DISCUSSION

To our best knowledge, this is the first study to systematically analyze the genotype distribution of three *MTHFR* polymorphisms (C677T, A1298C, and A1793G) and their associations with AAO, homocysteine, and WML in the Chinese AD cohort. We found two polymorphisms, including C677T and A1298C, were associated with AD in Chinese populations. Moreover, in *APOE4* carriers, the frequency of the allele T (C677T) was prominent in AD patients compared to controls, indicating that C677T might depend on the presence of *APOE4*.

Interestingly, the C677T polymorphism was associated with AAO, homocysteine, and WML, suggesting it may be a risk factor in developing AD. Several studies have documented that C677T resulted in a mildly dysfunctional thermolabile MTHFR enzyme, leading to reduced enzymatic activity and elevated blood homocysteine levels [19–21], suggesting it may play an important role in AD. In our results, we confirmed a significantly lower AAO in LOAD patients carrying CT/TT (C677T) genotypes, suggesting that C677T polymorphism was associated with susceptibility to LOAD. In contrast, AAO was significantly higher in LOAD patients carrying AC/CC (A1298C) and AG/GG (A1793G) genotypes, indicating A1298C and A1793G polymorphism may delay the onset of AD. Sutovsky *et al*. [22] reported that AAO in *APOE4* non-carriers was higher than *APOE4* carriers in AD patients (higher by 3.4 years), which was similar to our results (higher by 3.7 years in LOAD). Moreover, AAO in group of ‘*APOE* X/X + CC (C677T) + AA (A1298C)’ in AD patients was higher than group of ‘*APOE* 4/X + 4/4 + TT (C677T)’ or group of ‘*APOE* 4/X + 4/4 + CC (A1298C)’ (higher by 5.3 years and 2.9 years, separately). Combining the above two results, the single effect of TT (C677T) increased the difference in AAO (1.9 years), while CC (A1298C) decreased the difference in AAO (0.5 years), which was similar to our results (both 1.2 years in LOAD).

Consistent with previously reported results [23], the average level of homocysteine was significantly higher in TT (C677T) carriers in comparison with those of non-carriers (CC), suggesting that C677T polymorphism contributed to an elevated homocysteine level. In AC/CC (A1298C) and AG/GG (A1793G) carriers, the average level of homocysteine was lower in comparison with those of non-carriers (AA), but no statistical difference was determined. This trend indicated that the A1298C and A1793G might have an effect on lowering homocysteine. Moreover, the Fazekas score was higher in AD patients carrying CT/TT (C677T) genotypes, while the Fazekas score was higher in AD patients carrying AA (A1793G) genotype. These results showed that the C677T polymorphism was correlated with more severe WML, and A1793G polymorphism might contribute to milder WML. Like A1793G, the Fazekas score was higher in AD patients carrying AA (A1298C) genotype, while no statistical significance was determined. Considering that homocysteine and WML were determined by several factors in addition to *MTHFR* genotypes, including diet, lifestyle, and blood pressure [24, 25], more studies are required to confirm their associations in AD patients further.

There was a lack of association of the epistatic interaction between *APOE4* and *MTHFR* C677T polymorphism, either in LOAD or EOAD (*P* > 0.05), which was inconsistent with previously reported results [22, 26]. These may depend on diverse characteristics of the study population, including ethnicity and geographical location. The *APOE4* proportion was varied in different regions from 14.1% (lowest, Asia and Southern Europe) to 61.3% (highest, Northern Europe) [27]. The prevalence of *APOE4* alleles in our AD patients was 43.3% compared with 75.2% in the reported positive study (75.2%) [22], which may explain the difference. However, a significantly higher frequency of *MTHFR* CC genotype (A1298C) was observed in *APOE4* non-carriers in AD (*p* < 0.05), indicating that there may be an epistatic interaction between *APOE4* and *MTHFR* A1298C polymorphism.

The MTHFR enzyme is a dimer where each monomer is composed of a catalytic domain that binds the FAD cofactor and folate, and a regulatory domain that binds S-adenosylmethionine. The 222 position in the polypeptide (corresponding to 677 in cDNA) resides in the catalytic domain, and the 429 position (1298 in cDNA) resides in the regulatory domain [20]. Vraneković *et al*. [28] reported that the 'C configuration' (formed by genotypes of 677CC and 1298CC) of the MTHFR enzyme was more stable than the 'E configuration' (formed by genotypes of 677TT and 1298AA). Our various results of the C667T and A1298C may be explained by the destabilization of the enzyme dimer, which could be caused by the altered polypeptide conformations, including the movement of polypeptide domains induced by the variants [28, 29]. Inspired by the model of C667T and A1298C discussed above, we speculated that A1793G might function in a similar way, which requires further investigation. Moreover, the N5-methyltetrahydrofolate could spontaneously release formaldehyde (FA) after a series of actions [30]. Several studies provided evidence that endogenous formaldehyde was closely related to AD, such as essential for Aβ self-aggregation [31–33]. And scavenging FA could be an effective strategy for treating AD [31]. Therefore, polymorphisms of the *MTHFR* gene may affect the development of AD by affecting the release of FA.

## CONCLUSIONS

The genotype distributions of *MTHFR* C677T and A1298C polymorphisms are associated with AD in the Chinese population. Moreover, AD patients with C677T polymorphism are prone to present an earlier onset, higher homocysteine level, and more severe WML.

## Supplementary Materials

Supplementary Table 1
