# Supplementary material for: Genetic effect of MTHFR C677T, A1298C, and A1793G polymorphisms on the age at onset, plasma homocysteine, and white matter lesions in Alzheimer's disease in the Chinese population
Source: Aging (Albany NY). 2021 Apr 4;13(8):11352–62. doi: 10.18632/aging.202827 (PMC8109119; doi:10.18632/aging.202827)
Supplement: Supplementary Table 1 [file aging-13-202827-s001.pdf]

SUPPLEMENTARY TABLE

Supplementary Table 1. Primer sequence and annealing temperature for the *MTHFR* C677T, A1298C, and A1793G polymorphisms and APOE genotypes.

Position		Sequence 5′-3′	Annealing temperature (℃)
C677T	Forward	GAAAAGCTGCGTGATGATG	55
	Reverse	TTGAAGGAGAAGGTGTC	
A1298C	Forward	AAGAACGAAGACTTCAAA	55
	Reverse	TGGGGGGAGGAGCTGAC	
A1793G	Forward	TTGGAGAGCCCTGTTAATCTTG	58
	Reverse	AGAGACACGAAGGAGAGTGGAG	
APOE	Forward	TCCAAGGAGGTGCAGGCGGCGCA	65
	Reverse	ACAGAATTCGCCCCGGCCTGGTACACTGCCA	
